# A workflow to build PBTK models for novel species

**DOI:** 10.1007/s00204-020-02922-z

**Published:** 2020-10-09

**Authors:** Sebastian Schneckener, Thomas G. Preuss, Lars Kuepfer, Johannes Witt

**Affiliations:** 1 Applied Mathematics, Engineering and Technology, Bayer AG, Leverkusen, Germany; 2 Effect Modelling, Research and Development, Crop Science, Bayer AG, Monheim, Germany; 3 Systems Pharmacology and Medicine, Clinical Pharmacometrics, Bayer AG, Leverkusen, Germany

**Keywords:** PBPK, Physiologically-based pharmacokinetic modelling, PBTK, Cross-species extrapolation, OSP, Best practice workflow

## Abstract

Physiology-based pharmacokinetic and toxicokinetic (PBPK/TK) models allow us to simulate the concentration of xenobiotica in the plasma and different tissues of an organism. PBPK/TK models are therefore routinely used in many fields of life sciences to simulate the physiological concentration of exogenous compounds in plasma and tissues. The application of PBTK models in ecotoxicology, however, is currently hampered by the limited availability of models for focal species. Here, we present a best practice workflow that describes how to build PBTK models for novel species. To this end, we extrapolated eight previously established rabbit models for several drugs to six additional mammalian species (human, beagle, rat, monkey, mouse, and minipig). We used established PBTK models for these species to account for the species-specific physiology. The parameter sensitivity in the resulting 56 PBTK models was systematically assessed to rank the relevance of the parameters on overall model performance. Interestingly, more than 80% of the 609 considered model parameters showed a negligible sensitivity throughout all models. Only approximately 5% of all parameters had a high sensitivity in at least one of the PBTK models. This approach allowed us to rank the relevance of the various parameters on overall model performance. We used this information to formulate a best practice guideline for the efficient development of PBTK models for novel animal species. We believe that the workflow proposed in this study will significantly support the development of PBTK models for new animal species in the future.

**Electronic supplementary material:**

The online version of this article (10.1007/s00204-020-02922-z) contains supplementary material, which is available to authorized users.

## Introduction

Physiology-based toxicokinetic (PBTK) models, also called physiology-based pharmacokinetic (PBPK) models in pharmacology, are frequently used in toxicology and pharmacology to simulate xenobiotic concentrations in different body compartments. Generally, PBTK models allow a mechanistic description of physiological processes governing the absorption, distribution, metabolism and excretion (ADME) of substances with temporal and spatial (compartmental) resolution. A key property of the PBTK models is the utilisation of tissue–plasma partition coefficients, which allow the estimation of the concentration levels of a drug in different organs. PBTK models therefore provide a unique opportunity to specifically quantify drug exposure in different tissues, which ultimately determines the extent of a drug response (Bessems et al. 2014; Hartung et al. 2017; Thiel et al. 2018). Due to the large level of prior physiological information that is already included in the base PBTK model structure, only little additional data is required to fully identify the model and to make predictions on the toxicokinetics as well as the resulting tissue-specific compound exposure (Jones et al. 2009; Kuepfer et al. 2016). This is even though drug exposure levels in the tissues may have a significant level of uncertainty since they are estimated from few basic physicochemical properties of a substance such as molecular weight or lipophilicity (Kuepfer et al. 2016; Niederalt et al. 2018). However, it is the only possibility to simulate on target drug exposure without further, potentially time-consuming measurements. In a PBTK model, the properties of the organism, i.e. its physiology or anthropometry, are separated from the properties of the compound, i.e. the physicochemistry of a molecule. In addition, active processes such as enzymatic reactions in metabolism or transporter-mediated uptake and secretion may be considered in PBTK models (Meyer et al. 2012), which may be derived from in vitro studies, extrapolated from information in similar species or identified from targeted PK data. This strict separation between the properties of the organism on the one hand and the properties of the compounds on the other allows the exchange of either the organism physiology or the physicochemistry of a drug to consider the disposition of the same compound in different organisms or, in turn, the disposition of different drugs in the same species. This is the reason why PBTK modelling is becoming increasingly popular for *in silico* trial simulations in clinical phases of pharmaceutical development programmes (Lippert et al. 2012) and for cross-species extrapolation and planning of first-in-man studies in preclinical research (Jones et al. 2006; Thiel et al. 2015). In particular, cross-species extrapolation is supported by the generic model structure of PBTK models where basically all physiological parameters can be modified or replaced to adjust for the physiology of a novel species. Consequently, a validated physiological PBTK model can be re-used for any compound with relatively limited efforts and without testing in living animals and as such is in full accordance with the 3R principles (Russell and Burch 1959). PBTK models are therefore a very promising tool to help achieving the goal of U.S. EPA to eliminate all mammalian testing by 2035 (Grimm 2019).

The mandatory pre-requisite for cross-species extrapolations is that a species-specific reference model is available and carefully validated. Here and in the following, we use the term “species model” to describe the merely physiological, substance-independent part of a PBTK model, i.e., excluding substance parameters, the application regime, and active processes. In contrast, the term “PBTK model” describes the full PBTK model including administration of a substance. Available and validated mammalian species models include human (Kuepfer et al. 2016; Willmann et al. 2003a), monkey (Willmann et al. 2007), minipig, dog (Willmann et al. 2010), mouse (Schenk et al. 2017; Thiel et al. 2015), rat (Willmann et al. 2003b), and a recently developed rabbit model (Mavroudis et al. 2018). For a broader applicability of PBTK models, it would clearly be desirable to have models for more species, e.g. for applications in mammalian environmental risk assessment.

In European environmental risk assessment for birds and mammals (tier 1), the end points of the most sensitive test species are compared to the exposure of certain “generic focal species” whose exposure (i.e. uptake of the chemical) is considered to be representative of all species potentially at risk, including a safety factor of 10 (acute effects) or 5 (reproductive effects) (European Food Safety Authority 2009).

While this approach is a pragmatic solution given the limited knowledge, there are several questions where additional information would be helpful for decision-making. For example, toxicity tests are usually performed with standard laboratory species, not with the focal species. However, the same dose may lead to significantly different effect levels in different species. This difference can be caused by different ADME properties of the substance in the body, i.e. by different toxicokinetics leading to different levels of exposure at the target site or by different physiological responses towards the exposure with an exogenous substance, i.e. the resulting toxicodynamics. It has been argued that toxicokinetics are often the main determinant in cross-species differences (Brinkmann et al. 2014; McElroy et al. 2011). This argument is in line with observations from clinical development programmes where an understanding of drug pharmacokinetics was also found to significantly increase success rates for market authorisation of new molecules (van der Graaf and Benson 2011).

In addition, the real exposure pattern is often time-variable, e.g. due to dissipation of the chemical in the environment or multiple applications of a plant protection product. In contrast, toxicity tests are usually performed with a constant exposure pattern, and the time-variable behaviour is considered in risk assessment using the assumption of a constant time-weighted average.

In the face of the above-outlined limitations, it is plausible that PBTK modelling will significantly support the application of computational concepts in environmental toxicology to achieve a more mechanistic understanding of cross-species differences, to support extrapolation of toxicokinetics to generic focal species or to predict the effect of real, time-variable exposure patterns.

However, the development and subsequent validation of a species model is usually very labour intensive because several steps, such as compiling physiological data, their integration into the structural model equations and their subsequent validation and curation, are inevitably iterative, which may explain the limited number of environmentally relevant and validated species models available to date.

Given the large number of model parameters, it is likely not feasible to identify every parameter from scratch for each new species model. Instead, pragmatic default assumptions need to be taken for certain parameters, e.g. adopting the values from other mammalian species or calculating the value by means of extrapolation to limit the overall effort necessary. This approach was successfully taken for the parameterisation of the rabbit model (Mavroudis et al. 2018).

Generalisation of the approach requires the systematic prior identification of the potentially most sensitive parameters in a PBTK model. This approach is needed, in particular, because many of the several hundred independent physiological parameters of a species model may be largely insensitive for most applications such that the exact numerical value is of minor relevance. In turn, there may be key parameters that largely govern the disposition of a substance within an organism and that require particularly accurate parameter identification. The goal of this paper is to discriminate between both subgroups of species parameters, group them into relevant PBTK parameter groups, and to use this information to formulate a guideline for the development of novel species models. Thus, the development of models for new animal species will be largely simplified such that the application of PBTK models in ecotoxicology will be significantly supported. Our work is also largely motivated by open access and open source concepts in systems biology, systems pharmacology and systems medicine (Lippert et al. 2019; Wolstencroft et al. 2015, 2017), which all stress the need for full transparency of computational tools and models. This implies in particular the application of best practice standards for model development and model qualification which are largely supported by full documentation and accessibility of the free and open PBPK software PK-Sim from the OSP software suite (OSP: open system pharmacology, https://www.open-systems-pharmacology.org). In that sense, the previously published rabbit PBPK model (Mavroudis et al. 2018) can be seen as a blueprint of our concept since it fulfils all requirements in terms of accessibility, reproducibility and qualification. Of note, the workflow applied is independent of the specific PBTK software used, but represents rather a generic concept of broad applicability for the identification of key model parameters, which can then be used to efficiently build PBTK models for novel species. In that regard, it should be noted that the quantitative results may be software and hence model dependent.

**Fig. 1:**
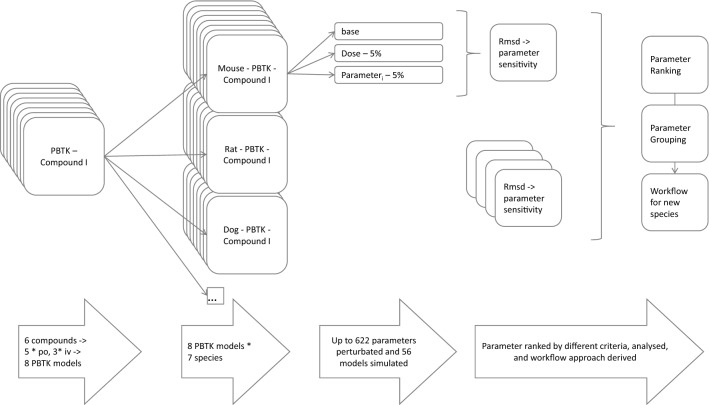
Graphical abstract of the approach to derive the best practice workflow

To identify the most sensitive parameters within the existing mammalian models, we first performed a cross-species sensitivity analysis. We extrapolated a set of validated PBTK models for rabbit to six additional mammalian species with established species models. Calculating the sensitivity over a range of different mammalian species as well as different compounds and different administration routes, we identified the most sensitive parameters for a large range of settings. We then used these findings to formulate a best practice workflow to guide the development of PBTK models for new animal species. The overall procedure to derive the workflow is summarised in Fig. 1.

## Materials and methods

### PBTK modelling

All PBTK models were built with PK-Sim^®^ from the open systems pharmacology software suite (OSP: https://www.open-systems-pharmacology.org, version 7.1.0) which is freely available as source code and executable, including a graphical user interface. All species models used are built into this open source software suite, so that all parameters and differential equations of the species models are accessible and editable. This includes in particular physiological parameters or the various species models (human Kuepfer et al. 2016; Willmann et al. 2003a), monkey (Willmann et al. 2007), minipig, dog (Willmann et al. 2010), mouse (Schenk et al. 2017; Thiel et al. 2015), rat (Willmann et al. 2003b) and rabbit (Mavroudis et al. 2018)).

### Rabbit reference PBTK models

Rabbit PBTK models have been previously developed for a set of marketed drugs, based on a standard rabbit species model (Mavroudis et al. 2018). The models were carefully informed and validated using PK data from the literature. Both i.v. and p.o. data were used if available. The consideration of multiple routes of administration is helpful for differentiating between drug distribution, metabolisation and excretion following i.v. dosing or drug absorption in the intestine for tablets. Data describing renal or faecal excretion were also taken into account in these models to ensure correctness of the underlying mass balances at the whole-body level. The rabbit PBTK models were used in the present study as a set of reference models for further cross-species extrapolation. Notably, the set of validated rabbit PBTK models allowed the equivalent consideration of physiological and physicochemical variables in the overall parameter space through cross-species extrapolation and subsequent sensitivity analyses.

### Cross-species extrapolation

Cross-species extrapolation of PBTK models was performed as described previously (Thiel et al. 2015). In brief, eight rabbit PBTK models for six different compounds were used as reference models for cross-species extrapolation to create the corresponding PBTK models for mouse, rat, dog, minipig, monkey and humans. PBTK modelling software tools usually provide a convenient platform for cross-species extrapolation because physiological parameters characterising the specific anatomy and physiology of the organism, such as organ volumes, blood perfusion rates or organ surface areas, are usually provided for humans and various laboratory animals in the PBTK modelling software. Hence, curated collections of physiological parameters are available to the user in customised databases. In particular, standard species models are available for the six additional mammalian species within PK-Sim and were used as a basis for scaling. During cross-species extrapolation, each physiological parameter of a reference species needs to be replaced by the parameter of the target species (Thiel et al. 2015). Some software tools, such as PK-Sim, facilitate this process by semi-automatic options for cross-species extrapolation. Note that the doses must be provided in relative amounts (mg/kg) to account for species-specific differences in body size and weight. After cross-species extrapolation, the simulations were visually checked for model correctness and simulation behaviour. Altogether, cross-species extrapolation of 8 rabbit PBTK models to 6 further mammalian species resulted in 56 PBTK models that were used for further analyses. All 56 PBTK models developed with this study are available from the authors upon request.

For species for which prior knowledge on physiological parameters is not available, an alternative option would be allometric scaling. Allometric scaling means that a model is scaled to a new species based on a target body weight (BW) by linearly scaling the organ weights to reach, in sum, the target body weight. The scaling of the gastrointestinal tract volume is based on the empirical scaling factor BW^1.06^ (Clauss et al. 2007). The dry matter intake (in mammalian herbivores) scales with BW^0.76^ (Clauss et al. 2007). Here, we suggest scaling the gastrointestinal tract surface with the same exponent. Lagos and Bozinovic present similar scaling exponents (Lagos and Bozinovic 1999). It should be noted that allometric scaling might be difficult to apply, e.g. when it comes to composite parameters such as peripheral venous blood (Thiel et al. 2015). For this reason, we considered prior information regarding physiological parameters whenever possible.

### Sensitivity analysis

The relevance of physiological PBTK model parameters was analysed in a systematic sensitivity analysis. To this end, 609 physiological model parameters referring to the properties of the organism were considered. Additionally, this implies that all compound-specific model parameters were neglected. A list of all analysed and excluded parameters is available in the Supplementary Information. Note that not all parameters exist in all models. For example, the rat has no gall bladder; hence, parameters relating to the gallbladder are missing in all rat models. Alternatively, all models for inulin are based on a distribution model that utilises cell pores for the transport of large molecules. Only the inulin models make use of this feature accordingly.

A normalised parameter sensitivity was estimated for each parameter: a model parameter *i* was perturbed, a concentration–time curve was simulated (*c*_*i*_(*t*)), and a root-mean-square deviation (RMSD) between the perturbed and standard concentration–time curve *c*_0_(*t*) was calculated. Then, the dose was changed by the same relative amount, and the simulated concentration–time curve (*c*_dose_(*t*)) was compared to the standard concentration–time curve *c*_0_(*t*) in terms of RMSD. The normalised parameter sensitivity sens_*i*_ is the ratio between the RMSD from parameter perturbation and the RMSD obtained for the adjusted dose, which was reduced by 5%. Concentration–time profiles in different tissues were used, in particular venous blood plasma and the intracellular space in the gonads and the brain. The latter two represent relevant examples for the prediction of organ-specific side effects (Pilari et al. 2017), henceforth referred to as tox organs. The parameters were ranked according to the maximum sensitivity that a parameter exhibited across all models, substances and application methods:

$${\text{PBTK}} \,\left( {{\text{params}}} \right) \to c_{0} \left( t \right),$$

$${\text{PBTK}}\, \left( {{\text{params}}, {\text{para}} \times 0.95} \right) \to c_{i} \left( t \right)$$

$${\text{PBTK}}\, \left( {{\text{params}}, {\text{para}}_{{{\text{dose}}}} \times 0.95} \right) \to c_{{{\text{dose}}}} \left( t \right){\text{PBTK}}\, \left( {{\text{params}}, {\text{para}}_{i} \times 0.95} \right) \to c_{i} \left( t \right),$$

$${\text{PBTK}} \left( {{\text{params}}, {\text{para}}_{{{\text{dose}}}} \times 0.95} \right) \to c_{{{\text{dose}}}} \left( t \right),$$

$${\text{sens}}_{i} = \frac{{{\text{rmsd}} \left( {c_{0} \left( t \right), c_{i} \left( t \right)} \right)}}{{{\text{rmsd}} \left( {c_{0} \left( t \right), c_{{{\text{dose}}}} \left( t \right)} \right)}}.$$

This metric of sensitivity was chosen such that it is robust across very different simulations. (a) It is robust against differences in simulation length. (b) The magnitude of exposure may depend on species and compound effects, it is normalised for. (c) The sensitivity is normalised for the effect of applied dose. This is achieved by a normalisation against a baseline time profile (*c*_0_(*t*)) and against the effect of dose change (*c*_dose_(*t*)). This facilitates the comparison of different compounds and different species model with each other.

## Results

**Fig. 2:**
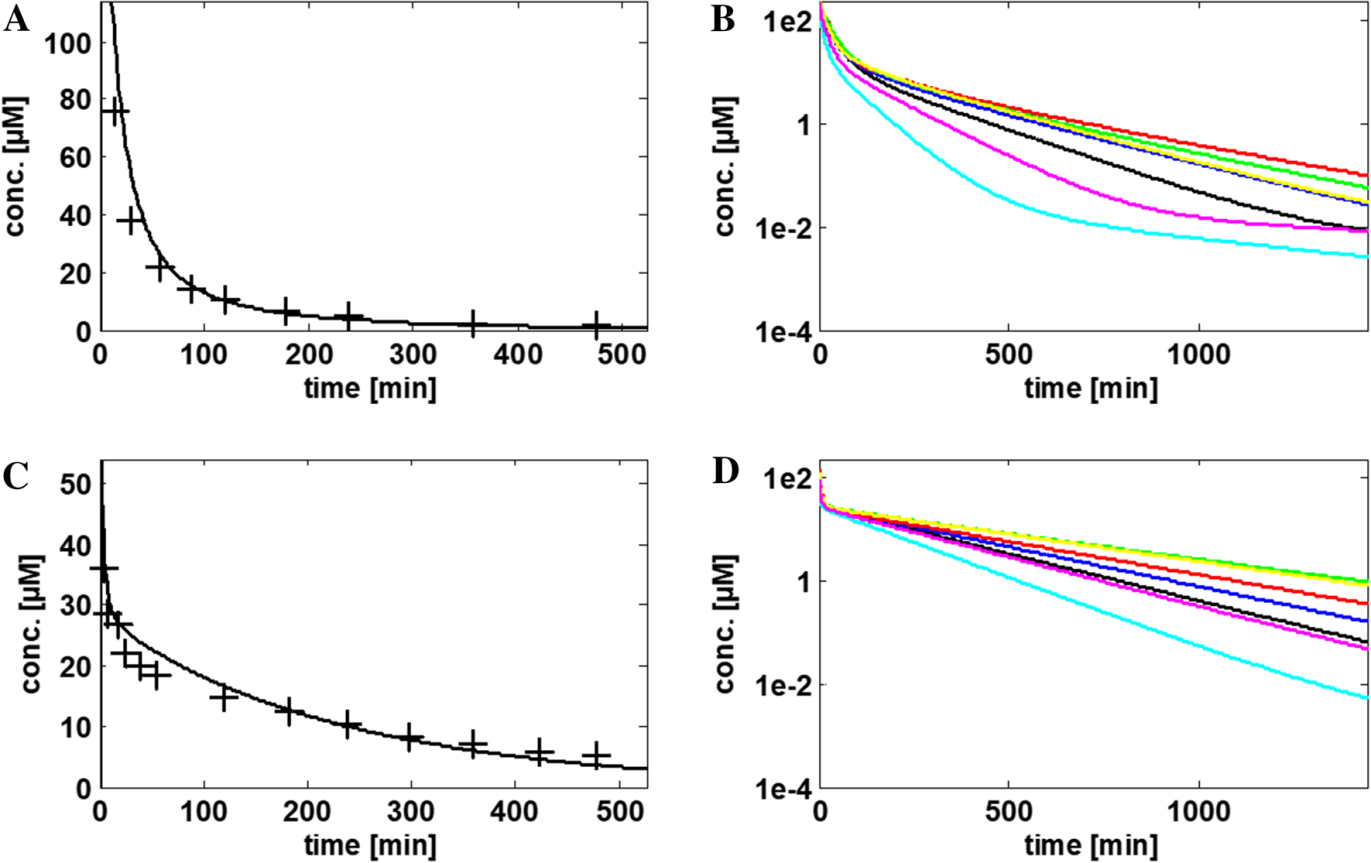
PBTK simulations for ofloxacin (**a**, **b**) and caffeine (**c**, **d**) in rabbits (**a**, **c**) (both intravenous administration) and seven mammalian species (**b**, **d**): rabbit (black), beagle (blue), human (red), minipig (green), mouse (cyan), monkey (yellow) and rat (magenta). PBTK simulations were performed with a previously published rabbit PBTK model (Mavroudis et al. 2018) and compared to experimental PK/TK data from Marangos et al. (1997) (**a**) and Beach et al. (1985) (**c**)

To formulate a best practice guideline for the development of new species models, we first aimed to categorise model parameters. In particular, we identified a set of key model parameters that largely govern the TK behaviour and that need to be set with utmost care to achieve accurate model simulations. To this end, we considered eight rabbit PBTK models [acyclovir (i.v. and p.o.), caffeine (i.v.), inulin (i.v.), ofloxacin (i.v.), paracetamol (p.o), and theophylline (i.v. and p.o.)], which were carefully validated previously (Mavroudis et al. 2018). Apart from inulin, all compounds represent typical small molecule compounds and have a drug metabolism with a similar complexity (molecular weight: 151–361 g/mol, inulin = 6179 g/mol; logP: − 0.02 to 1.25, inulin − 10). The selected compounds differ in clearance pathways, i.e. only glomerular filtration (inulin), only metabolic clearance (caffeine), tubular secretion and hepatic clearance (theophylline), and combinations thereof. The set of eight rabbit models was then used to generate the corresponding PBTK models of the same drug in mouse, rat, dog, minipig, monkey and humans through cross-species extrapolation (Jones et al. 2006; Thiel et al. 2015) (Fig. 2). In total, a set of 8 PBTK models for 7 model species was considered, resulting in a total set of 56 models (all 56 PBTK models are available upon request, see Materials and methods). The model-based translation is rather intuitive in PBTK modelling because it basically requires the systematic replacement of physiological parameters of a reference species (in this case, the rabbit) with those of the target species. Notably, these steps can be performed automatically in most PBTK software packages (see “Materials and Methods”). Additionally, doses were normalised with respect to body weight (mg/kg) to allow for a comparable amount of a drug that is administered in each case. This is of particular relevance because the body size and hence the corresponding weight varies significantly between mice (0.23 kg) and humans (73 kg).

The set of 56 PBTK models was used for a sensitivity analysis to identify key parameters governing species-specific ADME behaviour. Notably, only parameters involving species physiology were used in this step, while compound parameters were neglected. This differentiation between parameters of the organism and parameters of the compound was possible because the underlying model structure of PBTK models strictly separates both levels of input information (Kuepfer et al. 2016). This approach allowed us to analyse the physiological properties of the species without any potential influence of the compound. For the sensitivity analysis, 609 PBTK model parameters were considered for the set of 56 PBTK models (see “Materials and methods”), and their influence on the Cmax and AUC in different tissues was analysed. Thus, more than 100,000 sensitivities were estimated in total and further screened for their specific relevance.

### Relatively few parameters are sensitive

**Fig. 3:**
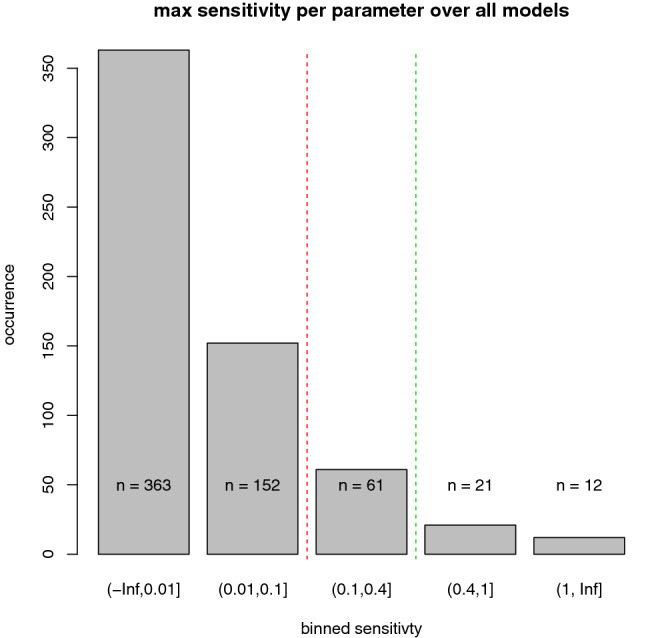
Histogram of the maximum sensitivities. A total of 609 different model parameters were analysed and binned according to the maximum estimated sensitivity over 56 different PBTK models. The majority of parameters had a sensitivity below 0.01, and 515 parameters had a sensitivity below 0.1 (85%). A total of 61 or 10% of the parameters had a moderate sensitivity between 0.1 and 0.4, while 33 (5%) parameters could be considered sensitive (> 0.4) in at least one model

To first rank the potential sensitivity of the parameters, the maximum sensitivity in plasma over all PBTK models was determined for each parameter. Figure 3 shows a histogram of the maximum sensitivities. A full list including parameter names and specific values is given in the Supplementary Information. It was found that most physiological model parameters have a low sensitivity across all models. In particular, we found that 363 (59.6%) of the parameters considered have a sensitivity below 0.01 in all investigated models and that 515 (84.6%) have a maximum sensitivity below 0.1 in all models. Typical examples of parameters with maximum sensitivity in plasma below 0.1 include the vascular fraction (in most compartments), the volume of smaller compartments such as the brain or heart, the interstitial fraction (in most compartments), and the hydraulic conductivity (in most compartments).

Only relatively few parameters (*n* = 33, 5.4%) have a sensitivity above 0.4 in at least one of the models. Most of these parameters (*n* = 20, 3.3%) are related to the gastrointestinal tract (GIT). These GIT parameters include the transit times, transit rates, pH, effective surface area enhancement factor, fractional steady-state fill level (of nutrients), gastric emptying time, length, and proximal and distal radii of different GIT compartments (Table 1). Sensitive parameters that are not related to the GIT include organ volumes of the liver, kidney and muscle; muscle tissue composition; interstitial, blood cells, and plasma composition (in terms of lipids, proteins, water); peripheral blood flow fraction (contribution from skin or muscle tissue blood); pH values (in the plasma or intracellular space); GFR (glomerular filtration rate); haematocrit values; specific blood flow rate in the kidney (volume flow rate per organ volume); and the fraction of the interstitial volume in skin (Table 2).

**Table 1:** GIT parameters with maximum sensitivity > 0.4

Text name	Explanation	Model name
pH (upper ileum)	pH of the respective GI segment lumen	Organism|Lumen|UpperIleum|pH
pH (lower ileum)		Organism|Lumen|LowerIleum|pH
pH (upper ileum)		Organism|Lumen|UpperIleum|Intestinal transit rate
pH (lower jejunum)		Organism|Lumen|LowerJejunum|pH
Effective surface area enhancement factor (lower jejunum)	Effective surface area enhancement factor of the respective GI segment	Organism|Lumen|LowerJejunum|Effective surface area enhancement factor
Effective surface area enhancement factor (upper ileum)		Organism|Lumen|UpperIleum|Effective surface area enhancement factor
Effective surface area enhancement factor (upper jejunum)		Organism|Lumen|UpperJejunum|Effective surface area enhancement factor
Effective surface area enhancement factor (lower ileum)		Organism|Lumen|LowerIleum|Effective surface area enhancement factor
Effective surface area enhancement factor (caecum)		Organism|Lumen|Caecum|Effective surface area enhancement factor
Proximal radius (lower jejunum)	Geometry of the respective GI segment	Organism|Lumen|LowerJejunum|Proximal radius
Distal radius (lower jejunum)		Organism|Lumen|LowerJejunum|Distal radius
Length (lower jejunum)		Organism|Lumen|LowerJejunum|Length
Intestinal transit rate (lower jejunum)	Transport rate through the respective GI segment	Organism|Lumen|LowerJejunum|Intestinal transit rate
Intestinal transit rate (upper ileum)		Organism|Lumen|UpperJejunum|Intestinal transit rate
Intestinal transit rate (caecum)		Organism|Lumen|Caecum|Intestinal transit rate
Transit time (large intestine)	Time for food to transit through the respective GI segment	Organism|LargeIntestine|Large intestinal transit time
Transit time (small intestine)		Organism|SmallIntestine|Small intestinal transit time
Gastric emptying time	Gastric emptying time	Organism|Lumen|Stomach|Gastric emptying time
Fractional steady-state fill level of nutrients (lower jejunum)	Fractional steady-state fill level of nutrients of the respective GI segment	Organism|Lumen|LowerJejunum|Fractional steady-state fill level
Fractional steady-state fill level (upper jejunum)		Organism|Lumen|UpperJejunum|Fractional steady-state fill level

**Table 2:** Non GIT-related parameters with maximum sensitivity > 0.4

Text name	Explanation	Model name
Liver volume	Organ volume	Organism|Liver|Volume
Kidney volume		Organism|Kidney|Volume
Muscle volume		Organism|Muscle|Volume
Specific blood flow rate (kidney)	Specific blood flow rate	Organism|Kidney|Specific blood flow rate
GFR (specific)	Glomerular filtration rate in kidney	Organism|Kidney|GFR (specific)
Tissue composition (muscle)	Tissue composition (lipids, proteins, water) of muscle	Muscle|Vf (eg. lipids)
Composition (organism)	Interstitial, blood cells, and plasma composition (in terms of lipids, proteins, water)	Organism|Vf (eg. lipids)
pH (plasma)	pH value	Organism|pH (plasma)
pH (intracellular)		Organism|pH (intracellular)
Haematocrit	Volume % of red blood cells	Organism|Haematocrit
Fraction interstitial (skin)	Fraction interstitial versus cells, plasma, and red blood cells in skin	Organism|Skin|Fraction interstitial
Peripheral blood flow fraction (muscle)	Contribution from muscle blood flow to peripheral blood flow	Organism|Muscle|Peripheral blood flow fraction
Peripheral blood flow fraction (skin)	Contribution from skin blood flow to peripheral blood flow	Organism|Skin|Peripheral blood flow fraction

In the single models, the number of sensitive parameters (sensitivity above 0.4) ranges from 1 to 4 for the i.v. models and from 2 to 18 for the p.o. models (Appendix). A total of 12 parameters showed sensitivities above 1 (2.50–1.05) in at least one model. Sensitivities above 1 occurred only in the p.o. models. Interestingly, this also included some parameters that were not from the GIT, such as the plasma pH or liver volume.

### Alternative metrics only have a limited effect on the qualitative results

When replacing our sensitivity metrics (based on rmsd) with metrics based on cmax, the parameters with maximum sensitivity above 0.4 were all contained in the list of sensitive parameters based on rmsd metrics. Only a few parameters with a maximum sensitivity above 0.4 for rmsd were not sensitive for *c*_max_ (Supplementary Information).

Furthermore, most parameters with a maximum (rmsd) sensitivity above 0.4 in plasma also have a maximum sensitivity above 0.4 in the brain or gonad tissue and vice versa*.* However, it should be noted that when investigating the sensitivity in brain or gonad tissue, parameters that are specific to this compartment (Vf, volume, specific blood flow rate) may also become sensitive (Supplementary Information).

### Sensitivity is qualitatively comparable across species for the non-GIT parameters but not always for the GIT parameters

**Fig. 4:**
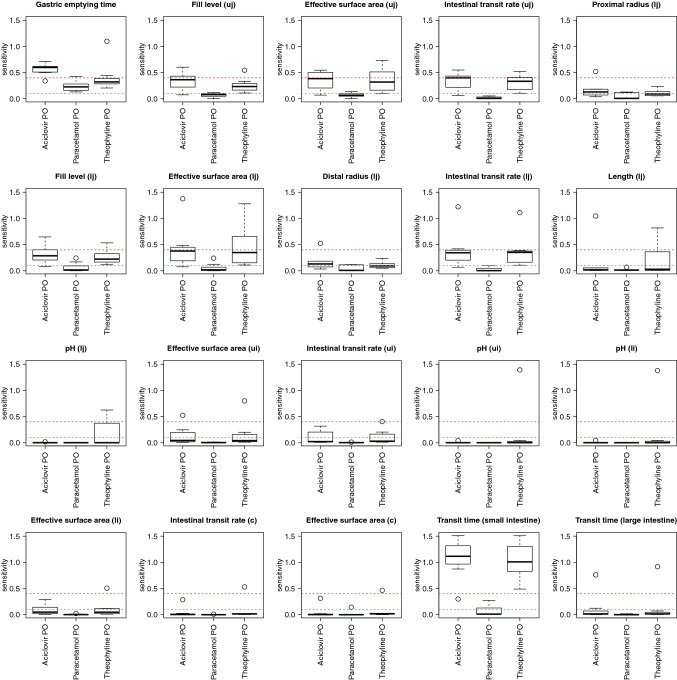
Variability of the GIT parameter sensitivity over species and substances (only p.o. administration). Each sub-plot represents one GIT model parameter with a sensitivity > 0.4 in at least one model. In each sub-plot, the *y*-axis indicates the sensitivity values for a particular substance/application combination (*x*-axis). The sensitivity values are depicted as box whisker plots and represent seven different species. The sensitivity may be highly variable across species—many GIT parameters are non-sensitive in one species but very sensitive in another. Single outliers in the sensitivity plots can be attributed to three different species, i.e., human, rat, and mouse. *ui* upper ileum, *li* lower ileum, *uj* upper jejunum, *lj* lower jejunum, *c* caecum)

Species-specific variability of the sensitivity was investigated for the parameters with maximum sensitivities above 0.4. For the GIT parameters (Fig. 4), the sensitivity may be highly variable across species; for example, many GIT parameters are non-sensitive in one species but very sensitive in another. Single outliers in the sensitivity plots can be observed for 3 different species, i.e., human, rat, and mouse.

**Fig. 5:**
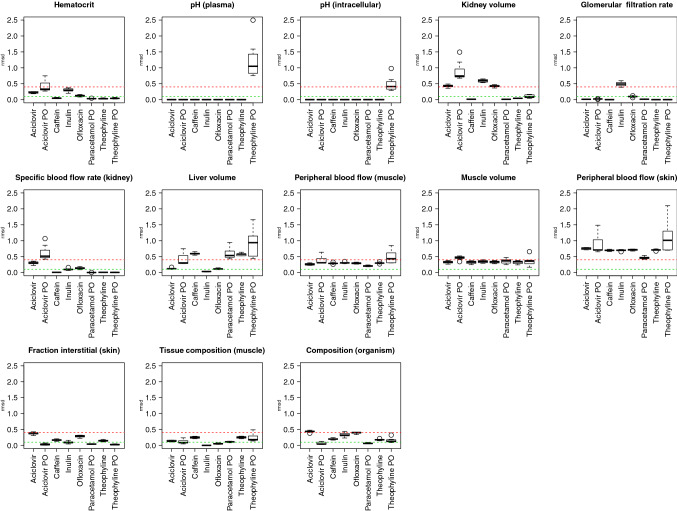
Variability of non-GIT parameter sensitivity over species and substances. Each sub-plot represents one non-GIT model parameter with a sensitivity > 0.4 in at least one model. In each sub plot, the y-axis indicates the sensitivity values for a particular substance/application combination (*x*-axis). The sensitivity values are depicted as box whisker plots and represent seven different species. It can be observed that in the majority of cases, the boxplot displays a narrow distribution of sensitivity values: the species-specific variability of parameter sensitivity is relatively small for non-GIT parameters over species, while between substances, the differences can be large

Species-specific variability is relatively small for non-GIT parameters in the i.v. models (Fig. 5), and some variability exists for the p.o. models, but without changing the qualitative assessment (e.g. as sensitive, moderately sensitive or insensitive). In contrast, the variability is often considerable across substances.

### Formulating a best practice guideline for PBTK model development

**Fig. 6:**
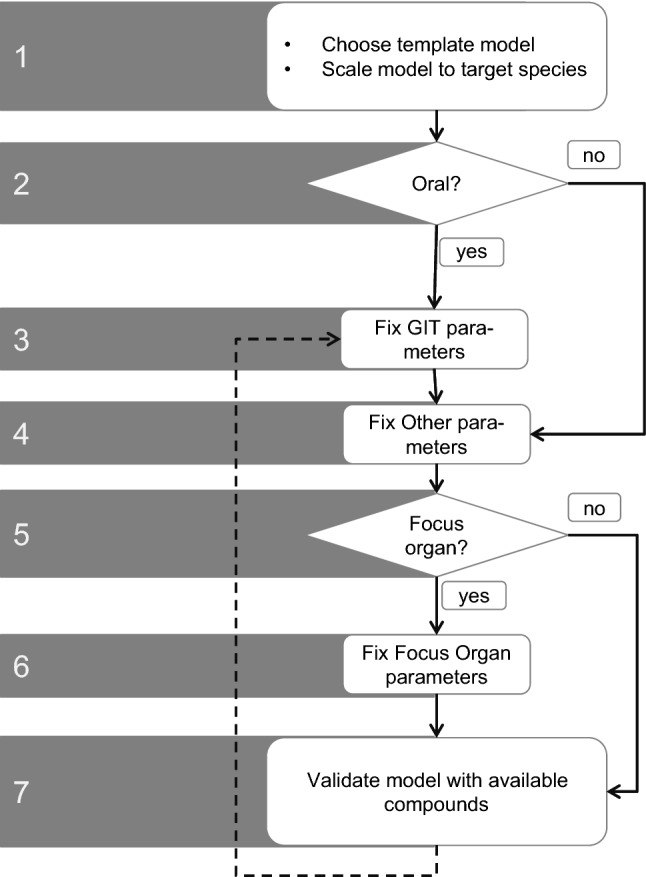
Best practice workflow for the PBTK model development

The above considerations can be used to formulate guidelines for the efficient development of the PBTK model for new species (Fig. 6). A main conclusion of the above sensitivity analyses is that GIT parameters are crucial to establish new species models. For example, GIT parameters such as the effective surface area enhancement factor or gastric emptying time showed large sensitivities and should be set with the utmost care, which means that these parameters should be subject to careful consideration either during literature review or by targeted experiments.

However, many of the parameters listed in Tables 1 and 2 are very difficult to quantify experimentally, or may jointly contribute to the description of the same process. We hence propose an additional functional categorisation of the identified parameters in functional groups of relevant PBTK parameters to further guide the required identification. Inspection of the GIT-related parameters in Table 1 shows for example (1) intestinal transit times, (2) intestinal pH, and (3) intestinal geometry as obvious umbrella terms for grouping. Likewise, (1) plasma parameters as well as (2) volume of distribution and clearance can be used for the categorisation of non-GIT parameters. It should be noted that the grouping of parameters may help to reduce the workload at this step and to focus the measurements on accessible parameters. If specific physiological model parameters are available for the target species, they should at any rate be used for the corresponding PBTK model. This may require a careful cross-check of model consistency as well as a potential adjustment of dependent parameters in some cases. However, such information represents valuable prior knowledge of highest significance.

An important question at this stage of model development is whether additional tox organs should be considered. If no tox organs need to be introduced, the initial PBTK model of a novel target species with the standard organs can be refined by generally setting species-specific parameters if available from a literature review, interspecies scaling or, ideally, one’s own experimental data. If a tox organ is to be additionally considered, species-specific parameters need to be set analogously (Pilari et al. 2017) (Table 3, third column); however, the parametrisation of the tox organ in novel species should be given particular attention to increase the accuracy of the computational predictions. Once the initial PBTK model of the target species is established, it should be iteratively qualified with PK data. The number of compounds is case specific and depends on a balance of uncertainty in simulation outcome, severity of the question at hand, and available resources. For example, in the previous development of a rabbit PBTK model (Mavroudis et al. 2018), PK data from six different compounds and eight datasets were used. In each case, the agreement between the simulations and the experimental results should be used to validate the model quality and to refine the model parameters. Here, the ranked list of sensitive model parameters may be used to focus on a set of highly relevant physiological parameters.

**Table 3:** Relevant PBTK parameter groups

GIT parameters	Non-GIT parameters	Tox organ parameters
Intestinal transit times Gastric emptying time Transit time (small intestine) Transit time (large intestine) Intestinal transit rate (caecum) Intestinal transit rate (upper ileum) Intestinal transit rate (lower jejunum) Intestinal pH pH (upper ileum) pH (lower ileum) pH (lower jejunum) Intestinal geometry Effective surface area (upper ileum) Effective surface area (lower ileum) Effective surface area enhancement factor (upper jejunum) Effective surface area (caecum) Effective surface area (lower jejunum) Length (lower jejunum) Proximal radius (lower jejunum) Distal radius (lower jejunum)	Plasma parameters pH (plasma) pH (intracellular) Haematocrit Volume of distribution and clearance Liver volume Kidney volume Specific blood flow rate (kidney) Muscle tissue composition Muscle volume GFR (specific) Composition (organism)	Volume of the organ Specific blood flow rate of the organ Tissue composition of the organ

Taken together, these considerations allowed us to formulate a guideline for the development of PBTK models for novel species (Fig. 6):

1. An established template model is chosen from a species with similar physiology, e.g. similar weight. If oral administration is considered, the species should also have similar food spectra because this determines the properties of the gastrointestinal tract. The template model is initially allometrically scaled (see “Materials and methods” regarding allometric scaling).
2. Which route of administration (i.v. or p.o.) will be considered? If oral absorption is not of immediate interest, the GIT of the initial target PBTK can be maintained, and no changes in the parameters are necessary.
3. GIT parameters are an important driver of the bioavailability of orally applied substances. The parameters from the GIT parameter column of Table 3 should be identified in the literature or experimentally and included in the new PBTK model.
4. The non-GIT parameter column of Table 3 lists all the parameters beyond the GIT parameters that are of general importance for the model. These parameters should be identified in the literature or by experiments.
5. Are there specific organs of interest? For example, the concentration in the brain might be important to predict behavioural effects or the concentration in gonads to predict reproductive effects.
6. If there are specific organs of interest, those organs should be parameterised specifically. The tox organ parameter column of Table 3 lists the most relevant organ-specific parameters.
7. Validation. The new model should be validated by observations in that species. To achieve validation, PBTK simulations for a species should be compared to observed data in that species. If differences beyond a threshold (typically a factor of 2 between mean observation and model, depending on uncertainty and variability of the measurements) appear, the model should be inspected and possible sources for discrepancy identified.

## Discussion

Computational models are increasingly important in all areas of life science to support a quantitative understanding of physiological processes. Computational models are particularly useful for predicting the response of an organism towards exposure to an exogenous chemical, whether a therapeutic drug or a potentially harmful toxin. In preclinical research and toxicology, computational models may therefore be particularly helpful in reducing the number of animal sacrifices because they largely support the application of the 3R principles during experimental studies. Another important application of computational modelling in life science is the translation of knowledge between different experimental setups and extrapolation across various animal species, patient cohorts or treatment schedules. In this regard, PBTK models are a good example of the supportive role of computational modelling in animal studies given the large level of physiological detail on which these models are built. However, the application of PBTK in environmental toxicology is frequently hampered by the limited availability of specific species models. To overcome this limitation, we present a generic best practice workflow that may be used to develop PBTK models for new animal species (Fig. 6).

The starting point of the proposed cross-species extrapolations is the availability of a carefully validated PBTK model for a reference species that can then be used for extrapolation to a target species. Here, a carefully validated set of rabbit PBTK models (Mavroudis et al. 2018) was used.

For the sensitivity analysis, we assumed that the active processes in the target species are the same as in the reference rabbit model. This simplification is likely untrue due to the evolutionary divergence of biological processes between species, but it allowed us to focus systematically on a mere functional effect of an active, protein-mediated process without explicitly considering the underlying genomics. Moreover, the assumption ensures that the complexity of each species model is comparable in terms of its structure and the total number of model parameters. Thus, it was also possible to first simulate the concentration–time curve of a drug in one species and then consider the same drug in another species. Conversely, a different drug could be considered in the same species by mainly exchanging the drug physicochemistry. Note that a strict validation of the PBTK models in species other than rabbit is not required for the purpose of this study because the models only aim at producing concentration profiles for further analyses (Fig. 2) instead of predicting the real behaviour of substances.

The cross-species extrapolation is the initial step in the development of a novel species model and is ideally supported by the PBTK software itself because cross-species extrapolation basically requires a mere exchange of physiological parameters from previous collections of organism-specific parameters. While the basic PBTK model for the target species can thus be obtained with little effort, the subsequent reviewing and fine-tuning of the model parameters, which in total add up to several hundreds, may result in a fairly laborious challenge. To limit the efforts required for the reworking of PBTK model parameters for the target species, we propose a best practice workflow that may be used to focus on a set of key parameters with high sensitivities (Fig. 6).

The sensitivities have been independently identified from a set of 56 PBTK models in seven animal species. Sensitivities were calculated as dose-dependent sensitivities, i.e., a change in a pharmacokinetic metric due to a change in a model parameter of 5% was normalised by the observed change in the same metric due to a dose reduction of 5%. Interestingly, it was found that most (84.6%) parameters had a relative sensitivity of below 0.1 throughout all models. In turn, only approximately 5.4% of parameters had a high sensitivity (above 0.4) in at least 1 of the 56 PBTK models considered.

While we suggest paying less attention to parameters with sensitivity below 0.1, they still might be relevant in special cases, though being compound specific. For example, consider a compound that accumulates in specific locations such that the parameterisation of this location becomes important.

Additionally, a number of parameters had a sensitivity greater than 1, meaning that modifying these parameters has an even higher impact than changing the dose. Interestingly, all observed cases from this set of parameters came from PO models. The reason for this high effect is dependent on the specific parameter, but can often be related to a change in bioavailability: drug absorption is saturated, and changing the dose has little effect on *c*_dose_(*t*). The denominator for calculating the sensitivity is therefore small, while changes in, e.g. the intestinal tract surface area increase the bioavailability and result in a relatively large numerator.

We used the identified set of key parameters to formulate our best practice guidelines for the development of PBTK models for novel animal species (Fig. 6). In particular, we provide relevant PBTK GIT parameter groups that need particular care during their identification, e.g. intestinal transit times, intestinal pH and intestinal geometry for oral administration (Table 3, and Table 1 for details). Among the non-GIT parameters, only two groups of parameters proved to be relevant, i.e. plasma parameters and volume of distribution and clearance (Table 3, and Table 2 for details). Plasma composition here determines the relative amount of lipids, proteins, and water which in turn governs drug distribution through passive and active transport processes. Still, it should be noted that some sensitive parameters may rather be neglected since they are largely similar in different species. For example, the pH of the plasma and intracellular space of different organs can be assumed to be comparable across mammalian species. Also, the peripheral blood flow fraction and interstitial fraction, while sensitive, are rather due to the specific model assumption that the plasma is sampled from superficial veins.

For identification of the more than 100,000 parameter sensitivities, we follow an unbiased and systematic approach including PBTK models of seven animal species. The sensitivity analyses in this study cover a wide range of the parameter space, including herbivorous, omnivorous and carnivorous species as well as a body weight span from 0.02 to 73 kg. Nevertheless, it cannot be excluded that further parameters would become sensitive if other species or other compounds were included in the analysis. For example, more lipophilic compounds than those tested here could result in a higher sensitivity for physiological parameters relating to fat tissue. The consideration of 56 PBTK models in seven animal species, however, gives confidence that key sensitivities and correlations were identified. Our work is based on very detailed implementation of PBTK models with many parameters. Another implementation of the PBTK approach in other software packages could result in a very different set of parameters, and surely different names for such parameters. The same holds if other partition models would be used (Kuepfer et al. 2016). In this case, the results can still be transferred by considering the semantics of the parameters, e.g. the volume of the liver is important here, because it influences metabolic clearance. An independent PBTK implementation could be void of a liver volume abstraction, but another parameter would be contributing to the extent of metabolic capacity and should be central for parameterisation. It should also be noted that all PBTK models developed in this study correspond to young adult animals or humans. If ageing was to be taken into account, species-specific changes in physiology had to be additionally considered (Schlender et al. 2016).

Our concepts largely rely on the full and unbiased accessibility of the underlying model structure which is increasingly advocated for in systems life sciences (Lippert et al. 2019; Wolstencroft et al. 2017, 2015). In that sense, transparency of the basic model equations and parameters is a mandatory pre-requisite of our approach. The recently published rabbit model (Mavroudis et al. 2018) fulfils all of these requirements in terms of documentation, qualification and reproducibility. The presented workflow represents a best practice guideline for the development of PBTK models for new animal species, which may be particularly useful for risk assessment in environmental toxicology and preclinical pharmacology. Another potential application of the workflow is the refinement of existing species models. In the previous development of PBTK models for different species, certain parameters have been assumed to be equal across species. If such parameters are actually sensitive model parameters, their values and their setting should certainly be reassessed.

The proposed workflow may hence also provide initial insights into the relevance of individual differences within one species; intra-species differences would be considered less relevant when they concern less sensitive parameters.

## Conclusions

In this work, we propose a best practice guideline for the development of PBTK models for new animal species. A preparatory sensitivity analysis in seven model species showed that only a few parameters are sensitive in each PBTK model. For the non-GIT parameters, the sensitivity is comparable across species, while GIT parameters may be substantially different in other species. However, substance-specific differences within one species are usually more pronounced than differences across species for the same substance. We believe that the workflow proposed in this study will significantly support the development of PBTK for new animal species, supporting the application of computational modelling in environmental toxicology.

## Electronic supplementary material

Below is the link to the electronic supplementary material.

Supplementary file1 (XLSX 1219 kb)
